# Spontaneous Dissection of the Renal Artery in Vascular Ehlers-Danlos Syndrome

**DOI:** 10.1155/2015/804252

**Published:** 2015-06-15

**Authors:** Filipa Pereira, Teresa Cardoso, Paula Sá

**Affiliations:** ^1^Serviço de Anestesiologia, Centro Hospitalar do Porto, Largo Professor Abel Salazar, 4099-001 Porto, Portugal; ^2^Unidade de Cuidados Intensivos Polivalente, Centro Hospitalar do Porto, Largo Professor Abel Salazar, 4099-001 Porto, Portugal

## Abstract

*Ehlers-Danlos syndrome* (EDS) is a rare heterogeneous group of connective tissue disorders. The vascular type (vEDS) is an autosomal dominant disorder caused by heterozygous mutations in the *COL3A1* gene predisposing to premature arterial, intestinal, or uterine rupture. We report a case of a 38-year-old woman with a recent diagnosis of vEDS admitted in the Emergency Department with a suspicion of a pyelonephritis that evolved to a cardiopulmonary arrest. A fatal retroperitoneal hematoma related with a haemorrhagic dissection of the right renal artery was found after emergency surgery. This case highlights the need to be aware of the particular characteristics of vEDS, such as a severe vascular complication that can lead to a fatal outcome.

## 1. Introduction

*Ehlers-Danlos syndrome* (EDS) is a rare heterogeneous group of connective tissue disorders [1]. The estimated prevalence varies between 1/5000 and 1/25000 [1, 2].

The* vascular type* (vEDS), formerly known as EDS type IV [3], is an autosomal dominant disorder caused by heterozygous mutations in the* COL3A1* [4]. Vascular EDS compromises 5–10% of all cases of EDS [4, 5]. The clinical hallmark of vEDS is excessive tissue fragility predisposing to premature arterial, intestinal, or uterine rupture, rarely observed in other forms of EDS [4, 6]. vEDS is also marked clinically by a characteristic facial appearance with decreased subcutaneous fat, a narrow nose, and thin lips and thin skin with visible superficial veins and prominent cutaneous bruising. Patients can also present premature aging of distal extremities (acrogeria) and joint hypermobility [5, 7]. However, these phenotype characteristics can be subtle and may not be diagnosed until postmortem examination [4, 5]. Furthermore, the natural course of vEDS and the clinical phenotype of patients are influenced by the type of COL3A1 variant [8].

A full differential diagnosis must be considered when the presenting signs and symptoms suggest vEDS. This includes* Marfan syndrome*,* Loeys-Dietz syndrome*, kyphoscoliotic types of EDS, idiopathic isolated arterial aneurysms, and autosomal dominant polycystic kidney disease. Clinical features, radiologic findings, and genetic characterization allow the clinical diagnosis and management [9–11].

Approximately 25% of patients with the vEDS experience the first complication by the age of 20, while 80% of patients have at least one complication by the age of 40 [6]. The median survival described in literature varies between 48 and 50 years [4, 6].

Treatment of complications associated with vEDS favours minimal noninvasive interventions. Invasive methods should be used only when necessary, primarily to save the patients' life [12].

We report a case of a 38-year-old woman with vEDS, who died during emergency surgery due to a severe vascular complication.

## 2. Case Presentation

A 38-year-old woman was admitted in the Emergency Department (ED) with a sudden onset of severe lower back pain, fever, nausea, and vomiting. The patient had a history of repeated urinary infections and renal lithiasis. She was being studied in the genetic department because of her history of infertility and spontaneous abortions and also because of the history of sudden deaths in her family (including her father). Therefore, she was diagnosed recently as having vEDS with a mutation in* COLA31* gene, but the specific defect was not mentioned in her genetic report.

On admission, her physical examination revealed translucid skin, thinned nose, and joint hypermobility of the lower limbs.

She had haemoglobin (Hg) of 13 g/dL, leukocytosis (16 × 10^9^/L), an elevated C-reactive protein (54 mg/dL), and inactive urinary sediment.

The renal ultrasound showed perirenal fluid and signs of nephritis but without obstruction.

**Figure 1:**
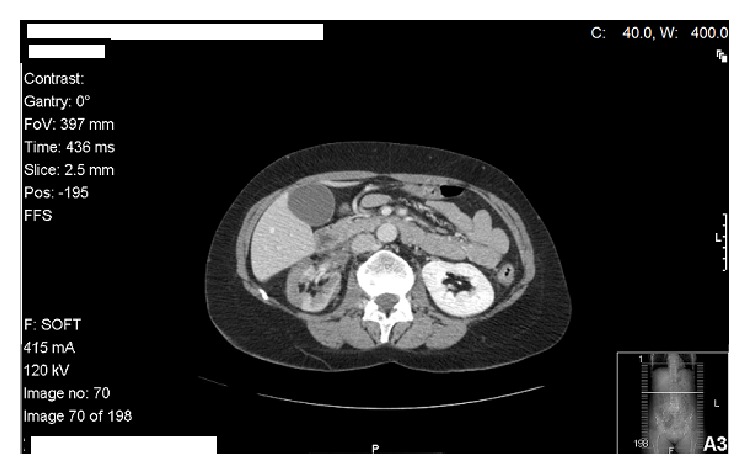
Abdominal Computer Tomography (CT) scan showing diffuse reduction in the parenchymal thickness of the right kidney and multiloculated fluid collection of about 10 centimetres (cm), involving the anterior part and the upper pole of the right kidney and the ipsilateral renal artery.

The Abdominal Computer Tomography (CT) scan (Figure 1) showed a diffuse reduction in the parenchymal thickness of the right kidney and multiple areas of* striatum* nephrogram, suggesting an inflammatory/infectious process (pyelonephritis). It also showed a multiloculated fluid collection of about 10 centimetres (cm), involving the anterior part and the upper pole of the right kidney and the ipsilateral renal artery, which appeared tapered and with a reduced calibre.

In between several exams, she was found unconscious in the clinical observation area of the ED and was taken to the Emergency Room (ER) in pulmonary arrest that rapidly evolved to cardiac arrest, from which she recovered with advanced life support in 10 minutes.

After initial stabilization in the ER, it was assumed that this clinical situation was due to a renal abscess, as shown in CT scan. A renal abscess drainage in the operating room was immediately proposed for her.

On arrival to the OR, she presented again in cardiopulmonary arrest in a nonshockable rhythm (pulseless electrical activity). Advanced life support (ALS) was started immediately.

The decision to perform an exploratory laparotomy, while in ALS manoeuvres, was taken and a team of vascular and urologic surgeons started together the procedure.

During surgery, a massive retroperitoneal hematoma was found, being largest at the left side. The infrarenal aorta was isolated but was found pulseless and empty. With the clamping of the supra- and infrarenal aorta no active haemorrhage was identified.

ALS manoeuvres were stopped 30 minutes after the beginning of surgery in the light of these findings and lack of any clinical response to massive transfusion measures.

Request for a clinical autopsy was obtained.

*Autopsy Findings*. The victim was a normally developed 38-year-old woman with apparent age superior to her real age. The skin of her knees was flaccid and pigmented and she had an articular hypermobility.

Internal findings included a hematoma in the top and posterior face of the right kidney which extended until the exterior of the renal capsule. There were multiple thrombosed renal vessels of medium and large calibre in the renal hilum, some of which presented a haemorrhagic dissection between the intimal and media arterial walls, sometimes with thrombosed pseudo lumens.

In all vascular territories the arterial walls presented morphological alterations of the intimal and media layers, namely, duplication and disruption of the internal elastic lamina, intimal nodular proliferation, and a media layer with thickening and thinning areas.

In These characteristics were suggestive of intimal and media fibromuscular dysplasia.

## 3. Discussion

Vascular EDS is a very challenging disorder. Therefore, it is important to suspect a severe vascular complication in a patient with a history of vEDS that presents in the ED with unspecific symptoms. These patients should be investigated promptly to rule out life-threatening situations due to vascular complications involving the vascular system, gastrointestinal tract, or gravid uterus [2, 6]. The vascular type of EDS describes a defect in the synthesis of type III procollagen and is the most severe form. Rupture of a previously nondilated artery is a common occurrence that results in considerable mortality and morbidity. Less commonly, an artery can dissect or dilate in either focal or diffuse form.

Therefore, patients with vEDS have a reduced life expectancy due to severe vascular complications. In a series of 31 patients, 61% present with at least one life-threatening vascular complication before the age of 40 [13].

An acute low back/flank pain is a common complaint in young adult presented to the ED and is usually due to a musculoskeletal or genitourinary tract cause. In our patient the exams showed an inflammatory/infectious process which was a likely diagnosis according to her past medical history of renal lithiasis. However, her recent diagnosis of vEDS was initially neglected and only because of a rapid hemodynamic deterioration was a surgical approach decided. There was a lack of communication among the medical staff, as the doctors responsible for the patient in the ED did not find her past medical history of vEDS important for this situation and this was not transmitted to the doctor responsible for the Emergency Room (ER) or to the radiologist responsible for the report of her abdominal CT scan. It is extremely important for radiologists to know if the patient has a collagen vascular disease when ordering the exam. There has to be more awareness about this syndrome among doctors and health staff working on the ED so that such situations can be minimized and a suspicious attitude could be adopted by every personnel that takes care of patients with vEDS. We have to remember that the incidence of this disease is very low (5% of all EDS patients) which means that most surgeons will never, or perhaps only once, meet a patient with the disease [12].

Patients with vEDS have unpredictable clinical courses and management is always challenging as they have high operative mortality rates. Therefore, we will never know if the course of our patient would have been different if a more suspicious attitude took place and all of the team responsible for the patient knew the disease and the complications involved. Nevertheless, we have to be aware of a severe vascular complication when receiving a vEDS patient in the ED.

A* disease specific passport* would be very helpful in these kinds of diseases where severe complications could be present and special attention and care are needed when these patients are in the ED. In the case reported, it would have been very helpful and could have alerted the physicians to have a more careful approach in patient's evaluation and care.

The autopsy revealed a retroperitoneal hematoma related with a haemorrhagic dissection of the right renal artery. This was probably due to the vascular malformation of fibromuscular dysplasia type. Although fibromuscular dysplasia is a different entity, patients with vEDS may have stenosis of renal and visceral arteries that mimic fibromuscular dysplasia. The diagnosis of these conditions relies on associated phenotypic traits and genetic tests: acrogeric dysmorphy, distal joint laxity, and tiny skin elasticity in vEDS, confirmed by detection of* COL3A1* gene mutations [14].

Regarding the treatment in vEDS, the most recent literature points to a rate of 29% mortality due to open and endovascular procedures less than the rates published in older studies [12, 13]. All invasive procedures should be avoided as far as possible as surgery can lead to vascular catastrophes and even death [13, 15]. A conservative approach or minimal invasive procedures should be preferred in these cases. Furthermore, the excessive rate of arterial complications in the past in endovascular procedures is believed to be related to large-diameter sheaths and devices used in prior decades. However, the prognosis after treatment is poor and invasive methods should be used only when necessary to save the patient's life [12].

There is no validated medical therapy in primary prevention of cardiovascular events in presymptomatic vEDS patients. The only medical therapy available is celiprolol, a *β*1-adrenoreceptor antagonist with a *β*2-adrenoreceptor agonist action. The only multicenter randomized trial by Ong et al., 2010, showed an effective prevention of major complications in patients with vEDS, with a reduction in arterial events (rupture or dissection) by three times compared with no treatment. Nevertheless, there is no indication that the benefit of celiprolol is a class effect and the question of beta blocker treatment remains open [16].

In summary, vEDS is associated with severe vascular complications in relatively young patients. We presented a case of a patient with vEDS with a fatal outcome and a misleading diagnosis after a spontaneous dissection of the renal artery.
